# Mutagenic consequences of a single G-quadruplex demonstrate mitotic inheritance of DNA replication fork barriers

**DOI:** 10.1038/ncomms9909

**Published:** 2015-11-13

**Authors:** Bennie Lemmens, Robin van Schendel, Marcel Tijsterman

**Affiliations:** 1 Department of Human Genetics, Leiden University Medical Center, Postzone S-4-P, P.O. Box 9600, 2300 RC Leiden, The Netherlands

## Abstract

Faithful DNA replication is vital to prevent disease-causing mutations, chromosomal aberrations and malignant transformation. However, accuracy conflicts with pace and flexibility and cells rely on specialized polymerases and helicases to ensure effective and timely replication of genomes that contain DNA lesions or secondary structures. If and how cells can tolerate a permanent barrier to replication is, however, unknown. Here we show that a single unresolved G-quadruplexed DNA structure can persist through multiple mitotic divisions without changing conformation. Failed replication across a G-quadruplex causes single-strand DNA gaps that give rise to DNA double-strand breaks in subsequent cell divisions, which are processed by polymerase theta (POLQ)-mediated alternative end joining. Lineage tracing experiments further reveal that persistent G-quadruplexes cause genetic heterogeneity during organ development. Our data demonstrate that a single lesion can cause multiple unique genomic rearrangements, and that alternative end joining enables cells to proliferate in the presence of mitotically inherited replication blocks.

## Abstract (web summary)

Barriers to DNA replication are potent sources of genome instability. Here, the authors provide a mechanistic model for how a single persistent G-quadruplex structure generates multiple substrates for polymerase theta-mediated end-joining, thus causing multiple deletions during animal development.

## Graphical abstract

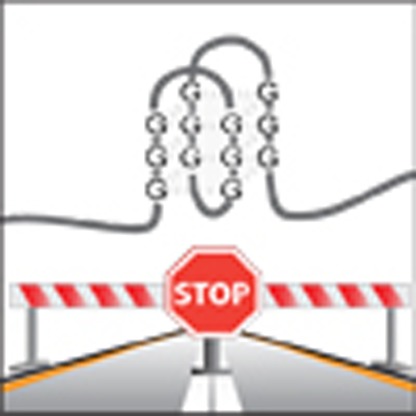

At present, surprisingly little is known about the molecular mechanisms that shape the genome during development and generate the mutations onto which natural selection can act. This lack of knowledge is mainly because of the very low frequency and the stochastic nature of spontaneous mutagenesis during unperturbed growth. Through next-generation sequencing of propagated cultures of *Caenorhabditis elegans*, we have recently shown that endogenous DNA lesions can cause severe genomic rearrangements during development^1^. A remarkably similar type of replication-associated mutagenesis is found at genomic sequences that are able to adopt G-quadruplexes^2^,^3^,^4^,^5^—thermodynamically highly stable DNA secondary structures that can form in G-rich single-stranded DNA (ssDNA). The rate of genome alterations at endogenous G4 motifs is, however, very low; even in animals genetically compromised for the G-quadruplex resolvase DOG-1/FANCJ, this rate is <0.1 per round of replication^2^,^3^,^4^,^5^.

Here we exploit the mutagenic nature and defined genomic position of these DNA secondary structures, to study the biological consequences of low frequent but potent replication blocks. We find G-quadruplexes to cause genomic deletions independent of the structure-specific nucleases MUS-81 and XPF-1. Instead, our data argues that these DNA structures escape active processing and persist during mitotic proliferation, imposing a new block to replication in each subsequent S-phase. In-depth mutation analysis and transgenic reporter studies indicate that a single replication-blocking lesion can generate multiple unique deletions among daughter cells, thereby fueling genetic heterogeneity during animal development.

## Results

### A model for mitotic inheritance of G-quadruplex structures

**Figure 1:**
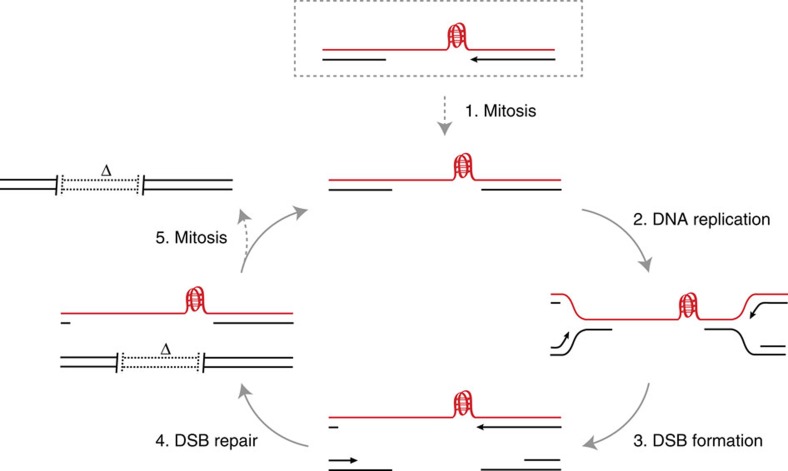
Model for mitotic inheritance of persistent G-quadruplexes. The upper illustration (dashed box) visualizes a replication-blocking G-quadruplex causing a ssDNA gap across the G-quadruplex-containing strand. In case quadruplexes are formed in the lagging strand, dsDNA 5′ to the G-quadruplex may mark the previously deposited Okazaki fragment. In case G-quadruplexes are formed during leading strand synthesis, dsDNA 5′ to the G-quadruplex may be the product of restart of replication downstream of the obstruction, or of an Okazaki fragment from a converging fork. 1. Mitotic inheritance of a stable G-quadruplex, paired with a gapped DNA strand, allows cycles of mutagenesis among proliferating cells (see step 2–5) 2. Similar to DNA interstrand cross-links, sporadic G-quadruplexes are expected not to impede overall genome duplication or the approach of converging forks, yet constitute a potent local block to nascent strand synthesis. 3. Failed replication of the G-quadruplex will generate a small ssDNA gap in the nascent strand, creating the pre-mutagenic lesion similar to the one before S-phase. However, replication of the gapped parental strand (heretofore located opposite the G-quadruplex) will cause a DSB. 4. Persistence of the G-quadruplex prevents DSB repair via HR, which requires templated DNA synthesis on the sister chromatid. Instead, the DSB is repaired via TMEJ, generating a small deletion in one of the sister chromatids. The resultant deletion mirrors the position and size of the ssDNA gap previously caused by the stable G-quadruplex. 5. Mitotic separation of sister chromatids generates two daughter cells, one inheriting a deletion and one inheriting a stable G-quadruplex and a ssDNA gap on that very same locus.

It has previously been proposed that G-quadruplex-induced genome alterations, which predominantly are deletions, arise because unresolved replication barriers trigger the formation of DNA double-strand breaks (DSBs)^3^,^6^. Structure-specific nucleases such as MUS81 and XPF have previously been implicated in DNA break formation at yet-undefined late-replication intermediates^7^,^8^,^9^,^10^; however, we found inactivation of MUS81 or XPF not to prevent G-quadruplex-induced mutagenesis, and also their combined inactivation did not influence the rate of G-quadruplex-induced deletion formation in *dog-1*-deficient animals (Supplementary Fig. 1). Previous work also excluded the requirement of nuclease scaffold protein SLX-4 or the Sgs1/BLM homologue HIM-6 for deletion formation at G4 motifs^11^,^12^. This lack of support for the involvement of endonuclease activity in processing G-quadruplexes made us address a more provocative hypothesis, in which a replication-blocking G-quadruplex is not resolved or processed. Instead, a G-quadruplex will repeatedly block replication during consecutive cell cycles and cause small ssDNA gaps that will be inherited to one of the daughter cells (Fig. 1). In this scenario it is the gapped DNA strand complementary to the strand harbouring the G-quadruplex that will generate a DSB, analogous to ssDNA nicks being converted into DSBs by DNA replication, while the replication barrier itself persists.

**Figure 2:**
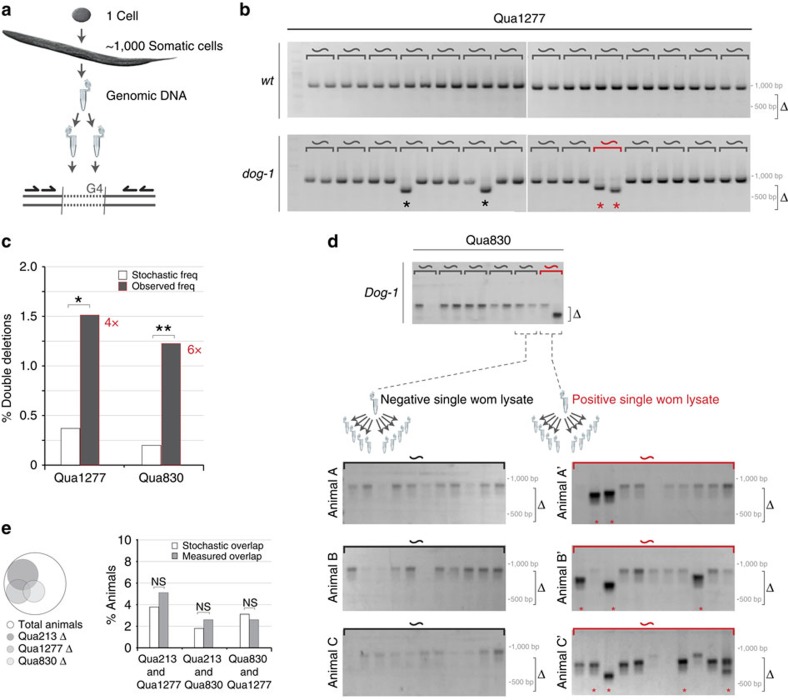
Overrepresentation of co-occurring G4 deletions in single animals. (**a**) Schematic representation of PCR-based experimental setup to identify G-quadruplex-induced deletions in single animals. (**b**) PCR analysis of G-quadruplex-induced instability at endogenous G4 site Qua1277 in *dog-1*-proficient (upper panel) and -deficient animals (lower panel). Representative gel images display the result of single animals (∼) assayed twice independently. See Supplementary Fig. 7a for additional gel images. The size range of PCR-amplified deletion products is indicated by Δ; two reference size markers (500 and 1,000 bp) are indicated; asterisks mark positive reactions/unique deletion products. (**c**) Quantification of the number of single animals that had two differently sized deletion (observed freq.), which was compared with the expected random double deletion frequency (white bars) based on frequency of deletions determined within the tested animal population (see Methods section for details). Asterisks indicate highly significant overrepresentation of the observed double deletion events within the tested population (*n=352 and **n=576) as determined by hypergeometric testing (*P<0.003 and **P<0.001). (**d**) PCR analysis of G-quadruplex-induced instability at endogenous G4 site Qua830 in *dog-1* animals, upper panel as in **b**. Single worm lysates were first categorized based on the presence or absence of a G4 deletion. Subsequently, the samples were assayed 11 times, to probe for the presence of additional G4 deletions. Three gel images representative for each category are depicted. Uncropped gel images are provided in Supplementary Fig. 7b. The size range of PCR-amplified deletion products is indicated by Δ; two reference size markers (500 and 1,000 bp) are indicated; asterisks mark uniquely sized deletion products (**e**) Venn diagram showing the distribution of G4 deletion events in 156 animals tested for all three loci. Histogram depicting the expected (white bars) and observed (black bars) frequency of animals showing a G4 deletion at both indicated G4 loci. NS indicates that the observed overlap does not statistically deviate from a random distribution, as determined by hypergeometric testing (*P*>0.20), which indicates that deletion events at different G4 loci are not interdependent.

To address this hypothesis we tested one of its predictions, which is that a single G-quadruplex, once formed, will spawn multiple deletions at the same genomic locus in descending cells (for a graphical illustration of this concept, see Supplementary Fig. 2). Individual *C. elegans* animals comprise ∼1,000 somatic cells, which are all derived from a single cell, the fertilized zygote. We reasoned that animals in which we detect a G-quadruplex-induced deletion should, based on the hypothesis above, be prone to have additional deletions at that very same locus (that is, in other daughter cells). Using a PCR-based strategy to detect genomic deletions at G4 loci (Fig. 2a), we indeed observed the frequency of additional deletions within affected animals to be profoundly higher than the stochastic deletion frequency in the population: for two typical endogenous G4 loci (that is, Qua830 on chromosome I and Qua1277 on chromosome IV) we found that individual animals frequently contained differently sized deletions (Fig. 2b, highlighted in red), with deletion frequencies within affected animals at least fourfold higher than the stochastic deletion frequency in the animal population (Fig. 2c). To verify this mutational bias towards affected animals (possibly bearing a G-quadruplex structure), we assayed limited dilutions of genomic DNA lysates of single animals, which, based on the first test, either had 0 or ≥1 deletions. Indeed, we detected numerous additional events in animals that had a deletion, whereas animals that were negative for the initial test rarely showed additional deletions (Fig. 2d and Supplementary Fig. 3). We found no correlation for deletion detection between the different G4 loci (Fig. 2e), suggesting that the mutational bias is locus specific and excluding the possible explanation that selected animals were more prone to deletion induction than others. The observation that some animals contain multiple deletions at the same genomic locus while the majority has none provides strong support for the existence and maintenance of a local pre-mutagenic substrate, which is not resolved, but instead persists during animal development, allowing it to repeatedly cause deletion-generating DSBs.

### A single unresolved G-quadruplex causes multiple deletions

**Figure 3:**
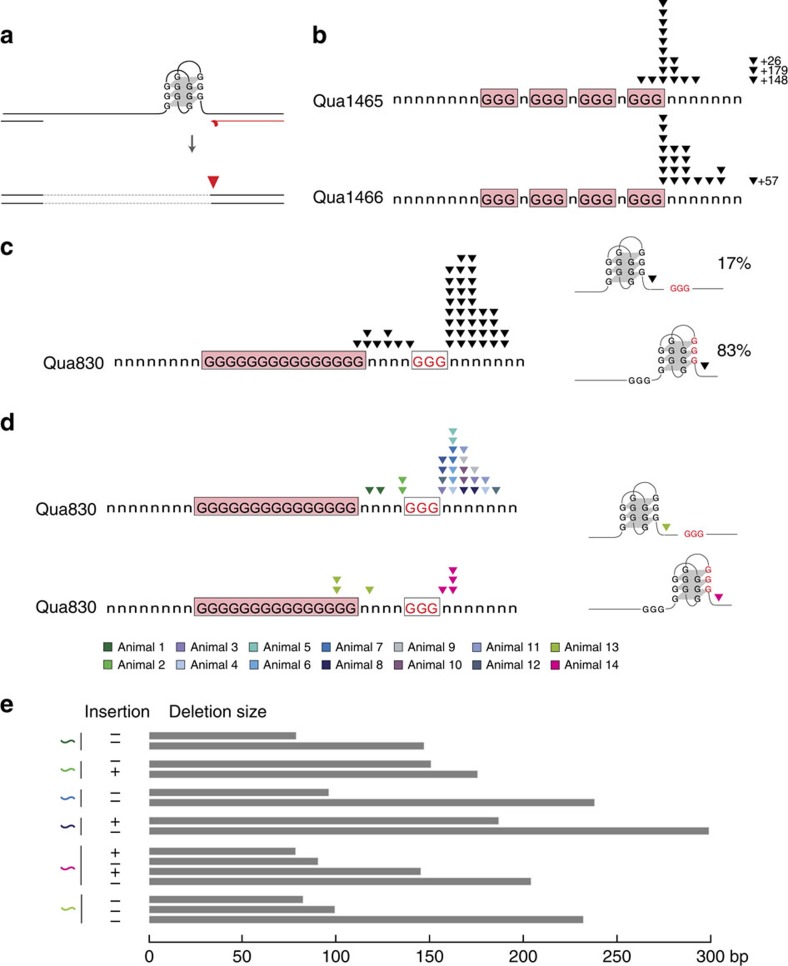
Signatures of inheritable G-quadruplex configurations. (**a**) Current model clarifying the correlation between the genomic position of a G4 motif and the 3′-junction of the cognate G-quadruplex-induced deletion: 3′-deletion junctions (red triangle) are defined by the halted progression of nascent strand synthesis at a G-quadruplex fold (red line). (**b**) Spectra of 3′-deletion junctions at indicated G4 loci. Each black triangle represents a 3′-deletion junction independently derived from single animals. (**c**) Bimodal spectrum of 3′-deletion junctions at Qua830. Black triangles represent 3′-deletion junctions independently isolated from individual animals. Illustrations on the right portray potential G-quadruplex configurations at Qua830 and the relative frequency of deletions, which, based on the position of their 3′-junctions, are attributed to that particular configuration. (**d**) Results of 3′-deletion junction analysis at Qua830 in single *dog-1*-deficient animals. The 3′-deletion junctions identified in 1% lysate fractions of the same individual are colour coded as indicated. Illustrations on the right portray G-quadruplex configurations at Qua830 and the position of recurrent 3′-deletion junctions identified in animal 13 and 14. (**e**) Graphical illustration of the G4 deletions described and depicted in **d**. The presence or absence of templated insertions is indicated (+/−, respectively).

To proof that all deletion events occurring within one animal were spawned by one single persistent DNA structure, we focused on the ability of a G-quadruplex to leave a unique genomic ‘scar’ that is indicative of its existence and of its configuration. We previously found the 3′-junctions of G-quadruplex-induced deletions to map immediately adjacent to the causative G4 motif^3^, possibly reflecting the collision of the replicative polymerase with a stable G-quadruplex (Fig. 3a). By analysing endogenous G4 motifs with only a single possible three-stacked G-quadruplex configuration, we further strengthened this notion: at two of such sites (Qua1465 and Qua1466) we found an extremely narrow distribution of 3′-junctions, with the vast majority (84% and 80%, respectively) mapping at or within 3 bp away from the first G of the G4 motif (Fig. 3b), which supports the explanation that the 3′-junction is defined by the inability of replicative polymerases to replicate across G’s that are within a G-quadruplex configuration^13^,^14^. The much wider distribution of 3′-junctions observed at G4 motifs that can adopt multiple G-quadruplex configurations (Supplementary Fig. 4 and refs 3, 4) may thus reflect several unique stalling events at a diversity of *in vivo* G-quadruplex folds. Analysis of another endogenous G4 locus, Qua830, further strengthened the correlation between G-quadruplex configurations and the 3′-junction of cognate deletions. Qua830 consists of a minimal G4 sequence and, separated by four nucleotides, an extra stretch of guanines that could either be entirely included or excluded in a G-quadruplex fold (Fig. 3c). We found two different populations of 3′-deletion junctions for this locus, one population mapping right next to the minimal G4 tract, the other mapping in front of the extra guanine triplet, thus >7 nucleotides away from the minimal G4 motif (Fig. 3c). This bimodal distribution of the 3′-deletion junctions implies two distinct G4 folding possibilities that are spatially sufficiently distinct to point towards two differently positioned replication blocks. It is this feature that provides the opportunity to experimentally test another prediction following from the hypothesis of heritable replication blocks: if a structure, once formed, will persist in a stable configuration and be inherited to daughter cells, it is predicted to block the replicative DNA polymerase at the very same genomic position in subsequent rounds of replication (Fig. 1). The predicted outcome is that deletions that occur within one animal will have very similarly positioned 3′-junctions, whereas deletions that occur independently in different animals will have unlinked 3′-junctions. This is exactly what we found. First, we assayed >300 single animals and analysed two different Qua830 deletions per individual, which when combined resulted in a bimodal distribution that was similar to the distribution derived from larger populations of animals (Fig. 3c,d, 17% of deletions mapped to the minimal G4 motif and 83% to the extended G4 motif). We found that all deletion pairs from individual animals (12/12) had matching 3′-deletion junctions and thus mapped to the same predicted G-quadruplex fold (Fig. 3d, binomial test: *P*<0.005). Next, we assayed limited dilutions of genomic DNA lysates of single animals to extract multiple deletion events and also found that all deletions occurring within one animal had matching 3′-junctions (Fig. 3d, bottom panel, binomial test: *P*<0.02). Together, these data strongly support the concept of a stable pre-mutagenic G-quadruplex that blocks DNA polymerase activity each successive round of replication, spawning multiple deletions at the same genomic location.

In sharp contrast to the 3′-junctions, the positions of the 5′-junctions of co-occurring deletions are seemingly randomly positioned, ∼50–300 bp away from the G4 motif (Fig. 3e). The variable but restricted distance to the G4 motif has led to the suggestion that this junction is defined by the deposition of Okazaki fragments^2^,^3^, either during lagging strand synthesis or through the action of a converging fork when leading strand synthesis is halted by a G-quadruplex, a scenario recently shown for replication-blocking cross-links^15^. A final feature of G-quadruplex-induced deletions, that is, the occasional presence of templated inserts, is also not influenced by common ancestry: we observed numerous cases of coupled deletions where only one of them contained a templated insert (Fig. 2e). It was recently demonstrated that templated inserts result from the action of polymerase θ during alternative, polymerase θ-mediated end joining (TMEJ) acting on G4-induced DSBs^3^; here we show that TMEJ repairs different DSBs that are being generated by the same heritable G-quadruplex.

**Figure 4:**
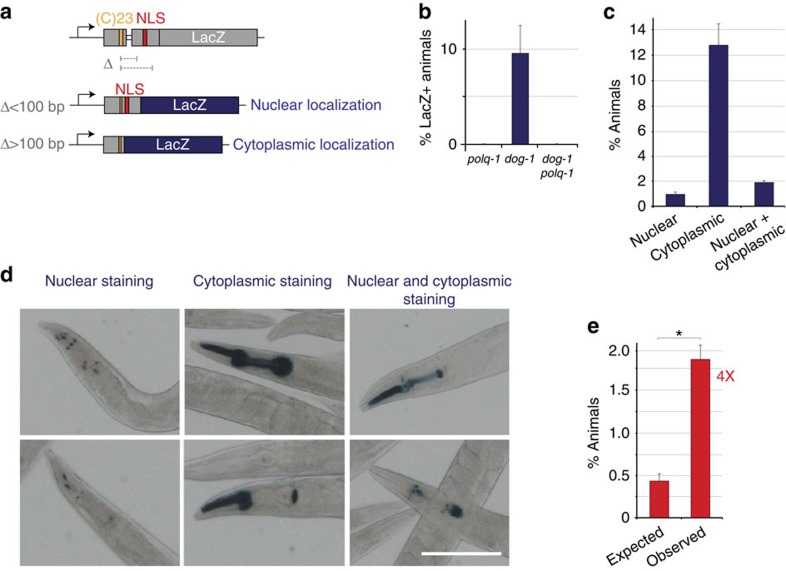
Inheritable mutagenic G-quadruplexes during organogenesis. (**a**) Schematic diagram of G4 instability reporter pLM88 and two categorically different TMEJ-generated repair outcomes. Although deletions <100 bp in size will retain the NLS in the LacZ ORF, deletions >100 bp will remove the NLS from the LacZ ORF. (**b**) Quantification of stochastic pLM88 ORF correction in asynchronous populations of the indicated genotype. Average percentage of LacZ-positive animals (*n*>180) of three independent experiments is depicted and error bars represent s.e.m. (**c**) Quantification of LacZ patterns in synchronized populations of *dog-1*-deficient animals transgenic for the G4 instability reporter (as illustrated in **d**). Average percentage of LacZ-positive animals of three independent experiments is depicted and error bars represent s.e.m. (**d**) Representative pictures of stochastic LacZ expression patterns of *dog-1*-deficient animals transgenic for the G4 instability reporter. Scale bar, 0.1 mm. (**e**) Comparison of the observed frequency of double events (as illustrated in **d**, right panels) and the expected frequency of such double staining patterns based on the frequency of the individual nuclear/cytoplasmic LacZ patterns. Asterisk indicates highly significant (*P*<0.001) overrepresentation of double events within the population (*n*=1,933) as determined by hypergeometric testing.

We next sought for *in vivo* validation of genetic heterogeneity resulting from heritable replication fork barriers. To this end, we constructed transgenic reporter animals to visualize G-quadruplex-induced deletions in individual cells. These animals express LacZ in pharyngeal muscle cells, but only when a G-quadruplex-induced deletion brings the open reading frame (ORF) in frame with the upstream ATG start codon (Fig. 4a). The *C. elegans* pharynx is a well-characterized organ and approximately ten mitotic divisions separate terminally differentiated muscle cells from the fertilized zygote. We hypothesized that a replication-obstructing G-quadruplex formed early in embryogenesis would trigger deletions that manifest in cells of the pharynx. To visualize different deletions in cells of one animal using only one read-out, that is, LacZ expression, we cloned a nuclear localization signal (NLS) 90 bp downstream of the G4 motif within a buffering sequence upstream of the LacZ ORF (Fig. 4a). Small deletions will produce NLS-containing ORFs and thus result in nuclear LacZ expression, whereas larger deletions remove the NLS and result in cytoplasmic expression. Indeed, *dog-1* deficiency resulted in a profound increase in LacZ-expressing pharyngeal muscle cells (Fig. 4b) and both nuclear and cytoplasmic staining patterns were observed (Fig. 4c,d), which, as expected, were fully dependent on functional TMEJ (Fig. 4b). We found 15% of animals to display cytoplasmic staining patterns and 3% to have nuclear LacZ staining patterns, in line with previous data showing that >75% of G4 deletions are larger than 100 bp (ref. 3). The frequency of animals that had nuclear and cytoplasmic LacZ expression, indicative of multiple unique G-quadruplex-induced deletions, was however significantly higher than expected based on the frequencies of the individual patterns (Fig. 4d,e). The observed fourfold increase is probably an underestimation of the actual number of animals suffering from coupled deletions, as this *in vivo* reporter system detects only in-frame deletion products and does not differentiate between deletions that are in the same size category. The notion that different deletions at the same locus frequently co-occur in somatic cells that share a common origin provides further support for the presence of inheritable replication blocks that persist during development, presenting a potent source of mutation and genetic mosaicism in somatic tissues.

## Discussion

Here we provide a model for how cells deal with physiological levels of replication obstacles and uncover the deleterious consequences of a single unresolved DNA secondary structure in developing tissues. Our data supports a model in which the failure to replicate across G-quadruplexed DNA results in a ssDNA gap that is transmitted to daughter cells. Such mitotic inheritance of a stable G-quadruplex, paired with a gapped DNA strand, allows for cycles of mutagenesis in proliferating cells: whereas next-round replication of the gapped parental strand results in a DSB, replication of the G-quadruplex will again generate a small ssDNA gap in the nascent strand, thus perpetuating the mutagenic cycle (Fig. 1). Error-prone repair of the resulting DSB through TMEJ causes deletions that in size reflect the length of the ssDNA gap (Fig. 1). Subsequent mitotic separation of sister chromatids generates two daughter cells, one inheriting this small deletion and one inheriting a stable G-quadruplex and a ssDNA gap at the corresponding genomic location.

One of the implications of imposing G-quadruplexes to persist throughout multiple cell cycles is that the replicative helicase is not able to unwind them: unwinding would either clear the impediment or allow it to refold before the approach of the replicative polymerases. However, we observed that multiple deletion events originating from one inheritable G-quadruplex all had junctions consistent with only one persistent configuration. The notion that deletion breakpoints map directly at or within 3 bp away from the first G of the G4 motif argues for DNA synthesis right up to the impediment. One explanation is that the MCM2-7 helicase cannot surpass a G-quadruplex obstruction and is disassembled before the approach of the replicative polymerases—such disassembly has been reported for replication-coupled repair of a DNA interstrand cross-link^16^, in which case DNA synthesis also advances to within one nucleotide of the cross-linked base. Alternatively, the Mcm helicase translocates past the G-quadruplex without disturbing it. Nakano *et al.*^17^ have shown that proteins smaller than 5 kDa when cross-linked to DNA do not block translocation of a ring-shaped heterohexamer consisting of human MCM4, 6 and 7, which thus may argue that it will also not necessarily be blocked by a 2- to 3-nm G-quadruplexed DNA knot. Future, *in vitro* work could help to resolve this question. Recent genetic data argues against the scenario of disassembly: BRC-1 is not influencing the frequency nor the pattern of G4-induced deletions^3^,^4^, whereas it was recently shown that *Xenopus laevis* BRCA1 promotes the dislocation of the MCM complex at replication-blocking interstrand cross-links^18^.

In this study we have focused on endogenous sequences that have the ability to adopt a G-quadruplex configuration to address the biological consequences of low-frequent replication fork barriers. Our findings, however, have a broader scope: we recently found that endogenous DNA lesions that stall DNA replication cause identical deletions and require the same alternative repair mechanisms^1^,^3^. This overlap is most parsimoniously explained by suggesting that also failed replication across a persistent DNA damage causes single-strand gaps that give rise to DSBs in subsequent cell divisions. Notably, animals do not have to be genetically compromised to manifest this type of genome instability at sites of blocked replication: small-sized deletions with distinct polarity can also be detected at G4 motifs in wild-type animals, albeit with extremely low incidence (10^−5^ per round of replication; Supplementary Fig. 5). Here we used *dog-1* deficiency to sensitize for G-quadruplex instability, as exposing animals to a G-quadruplex-stabilizing compound (pyridostatin) had no significant effect (Supplementary Fig. 6). With respect to cell type, we found that G-quadruplexes induce genome alterations in both somatic and germ line tissues^3^,^4^: using lacZ-based reporter transgenes we determined the rate of G-quadruplex instability in somatic tissue and in cells that contribute to the progeny, which, as such, define germ cells. When calculated per round of replication, both rates were similar. Finally, it is worth noting that G-quadruplexes also induce more extensive rearrangements, which in complexity and size resemble the complex rearrangements found in cancer cells^1^, and which are shown to be significantly enriched near G4 motifs in cancer tissues^19^. As these complex rearrangements are not visualized here (that is, the assays used have a ∼1 kb size constraint), our data probably underestimates the degree of genetic heterogeneity that result from persistent replication barriers. It is presently unknown whether the small-sized deletions and the more complex rearrangements are products of the same repair pathway.

We previously found that G-quadruplex-induced DSBs are repaired via an alternative, polymerase θ-mediated end-joining pathway^3^ that was recently shown to be conserved in mammals^20^,^21^,^22^. It was, however, not understood why these replication-associated DSBs were not repaired via homologous recombination (HR), as made evident by unaffected repair of G-quadruplex-induced DSBs in animals that are deficient for the essential HR factors BRC-1, BRD-1 or RAD-51 (refs 3, 4, 12). The data presented here provide an explanation for this pathway specificity. In case of a persistent replication block, the normal donor for HR, that is, the sister chromatid, cannot serve as a repair template, because it still harbours a replication-blocking structure (Fig. 1). In contrast, TMEJ does not require the sister chromatid and allows repair of DSBs when HR is (temporarily) hindered, albeit at the expense of small deletions. Such a critical back-up function of TMEJ provides a mechanistic basis for why HR-deficient (cancer) cells rely heavily on polymerase θ expression^23^,^24^,^25^.

## Methods

### Genetics

All strains were cultured according to standard *C. elegans* procedures^26^. Alleles used in this study include the following: *dog-1(gk10)*, *mus-81(tm1937)*, *xpf-1(e1487)*, *polq-1(tm2026)* and *IfIs77 [pLM88].*

### PCR-based assays to identify G4 deletions at endogenous loci

Stochastic deletion formation at endogenous G4 DNA loci was assayed using a PCR-based approach. Genomic DNA was isolated either from single worms or pools of worms and subjected to nested rounds of PCRs with primers that flank a G4 motif; all amplicons are >1 kb in size. PCR-based methods are highly sensitive and allows detection of low-frequency genomic rearrangements: G4 deletion products are preferentially amplified, because they are smaller than the abundant wild-type products and lack the G4 motif that hampers DNA replication *in vitro*^27^.

To capture independent G4 deletion events in individuals, L4 stage animals were used (one worm per 10 μl lysis reaction) and ∼0.1 μl lysate (1%) was transferred into 15 μl PCR reactions using a 384-pin replicator (Genetix X5050). Subsequently, 0.2 μl of PCR product was used for 15 μl nested PCR reactions. PCR reactions were typically run for 35 cycles with 54 °C primer annealing and 72 °C extension for 120 s. The following primers were used: Qua213: 5′-ctcagccaaggctacaaac-3′, 5′-gatacgtgtacatgaatagtc-3′, 5′-ccggcaattacacatttgcc-3′ and 5′-caaaactgtcgcctgacctc-3′; Qua1277: 5′-ggggagaagccgcatccaa-3′, 5′-cacatggagacggagagaaac-3′, 5′-cctgacaaacgcctactctc-3′ and 5′-gaatcccttttaatttggcaatag-3′; Qua830: 5′-ctagttcagggtatctggac-3′, 5′-ccttctctcgaagcgcgacc-3′, 5′-ggacggagagtcaataaaatc-3′ and 5′-cgaggtaaagtgcccgcaatc-3′; Qua1894 (previously named ggg317 (2): 5′-tttgccatcaaggttccaga-3′, 5′-ggatttcacagcgtcaagag-3′, 5′-tttgagccatataccaatc-3′, 5′-gtataagagttcctggtcggc-3′, 5′-cattgtgggaaaaatccgacg-3′ and 5′-aaaagtcgtgggaaaattg-3′. Deletion junctions were analysed by Sanger sequencing.

To obtain stochastic deletions frequencies at Qua1277 and Qua830, we analysed two independent PCR reactions on 1% lysate fractions of >190 *dog-1*-proficient and >330 *dog-1*-deficient L4 animals. Unique deletion products were discriminated based on size by gel electrophoreses. To obtain the expected frequency of animals carrying two independent stochastic deletions, we considered the two genomic lystate fractions as independent tests and multiplied the deletion frequency in the first sample to the frequency of unique deletions in the second sample. Dominant deletion products that resulted in identical deletions in both lysate fractions were assigned positive only in the first sample but not in the second, because these two deletion products did not represent independent stochastic events (see Supplementary Fig. 7a for an example). In fact, subsequent testing of single worm lysates that displayed two identical deletions resulted exclusively in identical PCR products (5/5), indicating that such deletion products act dominantly in the PCR reaction and probably prevent the amplification of independent stochastic events, in fact resulting in an underestimation of unique double deletion events. To determine whether there was significant overrepresentation of double deletion events, we used hypergeometric testing for overlap between the stochastic deletion frequency (based on the deletion frequency among independent individuals and size of the population) and the observed deletion frequency within the population sample carrying at least one unique deletion.

### LacZ-based reporter assay to visualize G4 deletions

Transgenic strains were obtained by microinjection of reporter construct pLM88 [myo-2::C23::stops::NLS::LacZ] and mCherry-based co-expression markers pGH8 and pCFJ104 (ref. 28), to generate *IfIs77*. To visualize stochastic G4 deletions, clonal lines of *dog-1*-deficient *IfIs77* animals were synchronized by bleaching and ∼200 L1 animals were grown on OP50 plates and stained for LacZ expression 3 days later^29^. To obtain LacZ expression frequencies, >4 synchronized populations of three independent clonal *dog-1 IfIs77* lines were analysed.

## Additional information

**How to cite this article:** Lemmens, B. *et al.* Mutagenic consequences of a single G-quadruplex demonstrate mitotic inheritance of DNA replication fork barriers. *Nat. Commun.* 6:8909 doi: 10.1038/ncomms9909 (2015).

## Supplementary Material

Supplementary Information Supplementary Figures 1-7 and Supplementary References.

## References

[b1] Roerink S. F., van Schendel R. & Tijsterman M. Polymerase theta-mediated end joining of replication-associated DNA breaks in *C. elegans*. Genome Res. 24, 954–962 (2014). 24614976 10.1101/gr.170431.113 PMC4032859

[b2] Cheung I., Schertzer M., Rose A. & Lansdorp P. M. Disruption of dog-1 in *Caenorhabditis elegans* triggers deletions upstream of guanine-rich DNA. Nat. Genet. 31, 405–409 (2002). 12101400 10.1038/ng928

[b3] Koole W. *et al.* A polymerase theta-dependent repair pathway suppresses extensive genomic instability at endogenous G4 DNA sites. Nat. Commun. 5, 3216 (2014). 24496117 10.1038/ncomms4216

[b4] Kruisselbrink E. *et al.* Mutagenic capacity of endogenous G4 DNA underlies genome instability in FANCJ-defective C. elegans. Curr. Biol. 18, 900–905 (2008). 18538569 10.1016/j.cub.2008.05.013

[b5] Pontier D. B. & Tijsterman M. A robust network of double-strand break repair pathways governs genome integrity during *C. elegans* development. Curr. Biol. 19, 1384–1388 (2009). 19646877 10.1016/j.cub.2009.06.045

[b6] van Kregten M. & Tijsterman M. The repair of G-quadruplex-induced DNA damage. Exp. Cell Res. 329, 178–183 (2014). 25193076 10.1016/j.yexcr.2014.08.038

[b7] Agostinho A. *et al.* Combinatorial regulation of meiotic holliday junction resolution in *C. elegans* by HIM-6 (BLM) helicase, SLX-4, and the SLX-1, MUS-81 and XPF-1 nucleases. PLoS Genet. 9, e1003591 (2013). 23901331 10.1371/journal.pgen.1003591 PMC3715425

[b8] O'Neil N. J. *et al.* Joint molecule resolution requires the redundant activities of MUS-81 and XPF-1 during *Caenorhabditis elegans* meiosis. PLoS Genet. 9, e1003582 (2013). 23874209 10.1371/journal.pgen.1003582 PMC3715453

[b9] Saito T. T., Lui D. Y., Kim H. M., Meyer K. & Colaiacovo M. P. Interplay between structure-specific endonucleases for crossover control during *Caenorhabditis elegans* meiosis. PLoS Genet. 9, e1003586 (2013). 23874210 10.1371/journal.pgen.1003586 PMC3715419

[b10] Minocherhomji S. & Hickson I. D. Structure-specific endonucleases: guardians of fragile site stability. Trends Cell Biol. 24, 321–327 (2014). 24361091 10.1016/j.tcb.2013.11.007

[b11] Saito T. T., Youds J. L., Boulton S. J. & Colaiacovo M. P. *Caenorhabditis elegans* HIM-18/SLX-4 interacts with SLX-1 and XPF-1 and maintains genomic integrity in the germline by processing recombination intermediates. PLoS Genet. 5, e1000735 (2009). 19936019 10.1371/journal.pgen.1000735 PMC2770170

[b12] Youds J. L., O'Neil N. J. & Rose A. M. Homologous recombination is required for genome stability in the absence of DOG-1 in *Caenorhabditis elegans*. Genetics 173, 697–708 (2006). 16547095 10.1534/genetics.106.056879 PMC1526509

[b13] Castillo Bosch P. *et al.* FANCJ promotes DNA synthesis through G-quadruplex structures. EMBO J. 33, 2521–2533 (2014). 25193968 10.15252/embj.201488663 PMC4282361

[b14] Kumari R., Nambiar M., Shanbagh S. & Raghavan S. C. Detection of g-quadruplex DNA using primer extension as a tool. PLoS ONE 10, e0119722 (2015). 25799152 10.1371/journal.pone.0119722 PMC4370603

[b15] Zhang J. *et al.* DNA interstrand cross-link repair requires replication-fork convergence. Nat. Struct. Mol. Biol. 22, 242–247 (2015). 25643322 10.1038/nsmb.2956 PMC4351167

[b16] Raschle M. *et al.* Mechanism of replication-coupled DNA interstrand crosslink repair. Cell 134, 969–980 (2008). 18805090 10.1016/j.cell.2008.08.030 PMC2748255

[b17] Nakano T. *et al.* Translocation and stability of replicative DNA helicases upon encountering DNA-protein cross-links. J. Biol. Chem. 288, 4649–4658 (2013). 23283980 10.1074/jbc.M112.419358 PMC3576070

[b18] Long D. T., Joukov V., Budzowska M. & Walter J. C. BRCA1 promotes unloading of the CMG helicase from a stalled DNA replication fork. Mol. Cell 56, 174–185 (2014). 25219499 10.1016/j.molcel.2014.08.012 PMC4185004

[b19] De S. & Michor F. DNA secondary structures and epigenetic determinants of cancer genome evolution. Nat. Struct. Mol. Biol. 18, 950–955 (2011). 21725294 10.1038/nsmb.2089 PMC3963273

[b20] Kent T., Chandramouly G., McDevitt S. M., Ozdemir A. Y. & Pomerantz R. T. Mechanism of microhomology-mediated end-joining promoted by human DNA polymerase theta. Nat. Struct. Mol. Biol. 22, 230–237 (2015). 25643323 10.1038/nsmb.2961 PMC4351179

[b21] Yousefzadeh M. J. *et al.* Mechanism of suppression of chromosomal instability by DNA polymerase POLQ. PLoS Genet. 10, e1004654 (2014). 25275444 10.1371/journal.pgen.1004654 PMC4183433

[b22] Zahn K. E., Averill A. M., Aller P., Wood R. D. & Doublie S. Human DNA polymerase theta grasps the primer terminus to mediate DNA repair. Nat. Struct. Mol. Biol. 22, 304–311 (2015). 25775267 10.1038/nsmb.2993 PMC4385486

[b23] Chan S. H., Yu A. M. & McVey M. Dual roles for DNA polymerase theta in alternative end-joining repair of double-strand breaks in *Drosophila*. PLoS Genet. 6, e1001005 (2010). 20617203 10.1371/journal.pgen.1001005 PMC2895639

[b24] Mateos-Gomez P. A. *et al.* Mammalian polymerase theta promotes alternative NHEJ and suppresses recombination. Nature 518, 254–257 (2015). 25642960 10.1038/nature14157 PMC4718306

[b25] Ceccaldi R. *et al.* Homologous-recombination-deficient tumours are dependent on Poltheta-mediated repair. Nature 518, 258–262 (2015). 25642963 10.1038/nature14184 PMC4415602

[b26] Brenner S. The genetics of *Caenorhabditis elegans*. Genetics 77, 71–94 (1974). 4366476 10.1093/genetics/77.1.71 PMC1213120

[b27] Pontier D. B., Kruisselbrink E., Guryev V. & Tijsterman M. Isolation of deletion alleles by G4 DNA-induced mutagenesis. Nat. Methods 6, 655–657 (2009). 19684597 10.1038/nmeth.1362

[b28] Frokjaer-Jensen C. *et al.* Single-copy insertion of transgenes in *Caenorhabditis elegans*. Nat. Genet. 40, 1375–1383 (2008). 18953339 10.1038/ng.248 PMC2749959

[b29] Pothof J. *et al.* Identification of genes that protect the *C. elegans* genome against mutations by genome-wide RNAi. Genes Dev. 17, 443–448 (2003). 12600937 10.1101/gad.1060703 PMC195995

