# Supplementary material for: Mutagenic consequences of a single G-quadruplex demonstrate mitotic inheritance of DNA replication fork barriers
Source: Nat Commun. 2015 Nov 13;6:8909. doi: 10.1038/ncomms9909 (PMC4654259; doi:10.1038/ncomms9909)
Supplement: Supplementary Information — Supplementary Figures 1-7 and Supplementary References. [file ncomms9909-s1.pdf]

**Supplementary Figure 1**

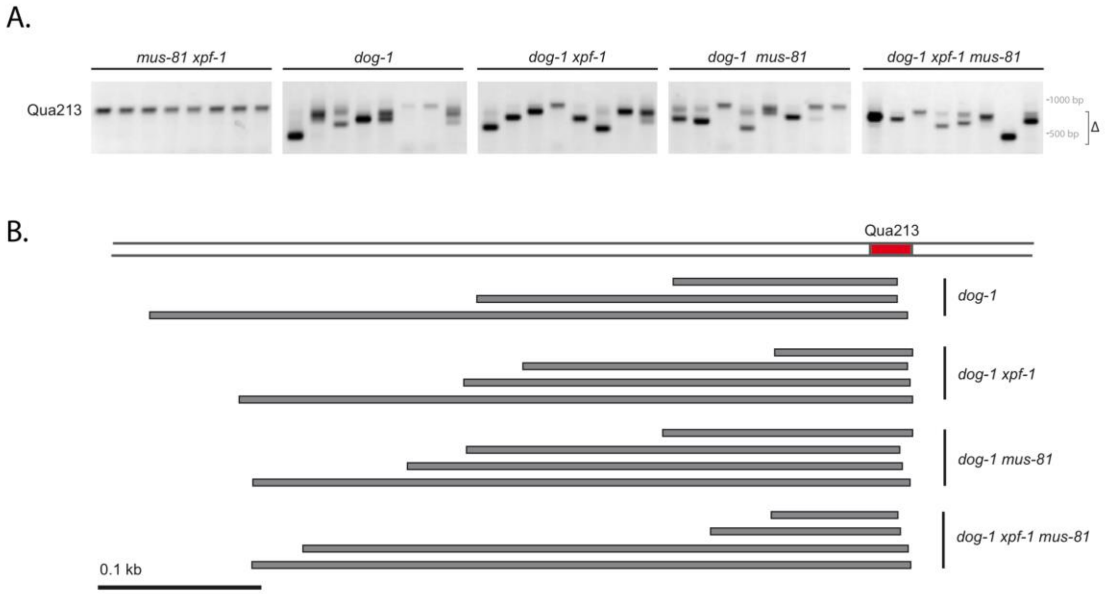


**Supplementary Figure 1. MUS-81 and XPF-1 are dispensable for deletion formation at G4 sites.** **A.** PCR analysis of G4 instability at endogenous G4 site Qua213; each well represents an independent PCR reaction on 10% single worm lysate. The size-range of PCR-amplified deletions products is indicated by Δ; two reference size markers (500bp and 1000bp) are indicated. **B.** Examples of G4 deletions identified in the indicated genetic backgrounds. Deletion analysis was performed on second-generation mutants to avoid maternal contribution of nuclease activities.

**Supplementary Figure 2**

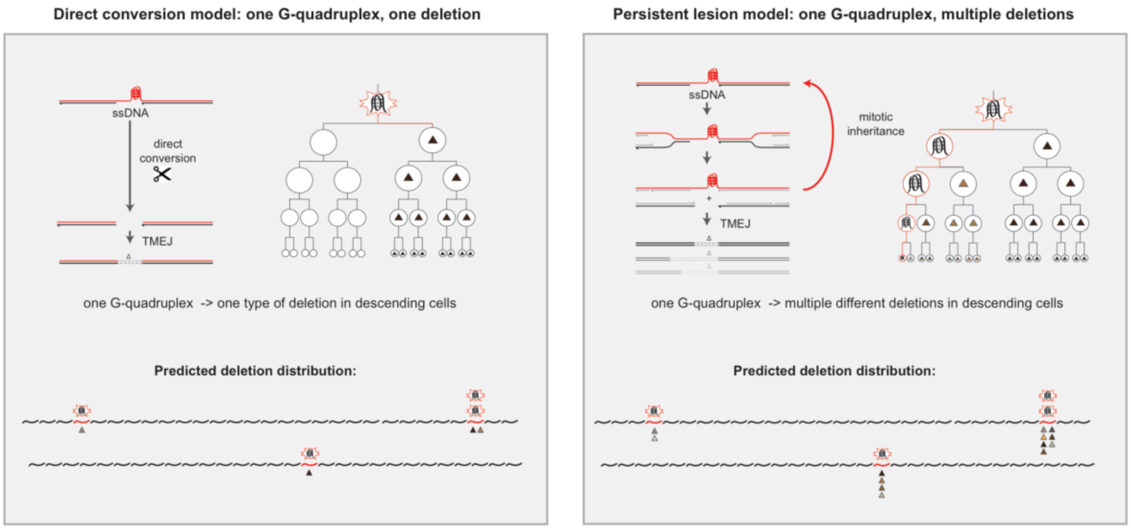


**Supplementary Figure 2. Hypothetical models for G-quadruplex-induced deletion formation and their predicted impact on G4 deletion distribution.**

**Left:** A "direct conversion model" where a mutagenic G-quadruplex is processed and thus lost during deletion formation, resulting in a random distribution of unique deletions among individuals and hence a relative low probability of multiple unique deletions in single animals. **Right:** A "persistent lesion model" where a pre-mutagenic G-quadruplex persists across multiple cell divisions spawning differently sized deletions in descending cells. This scenario results in an overrepresentation of multiple unique deletions in single animals.

**Supplementary Figure 3**

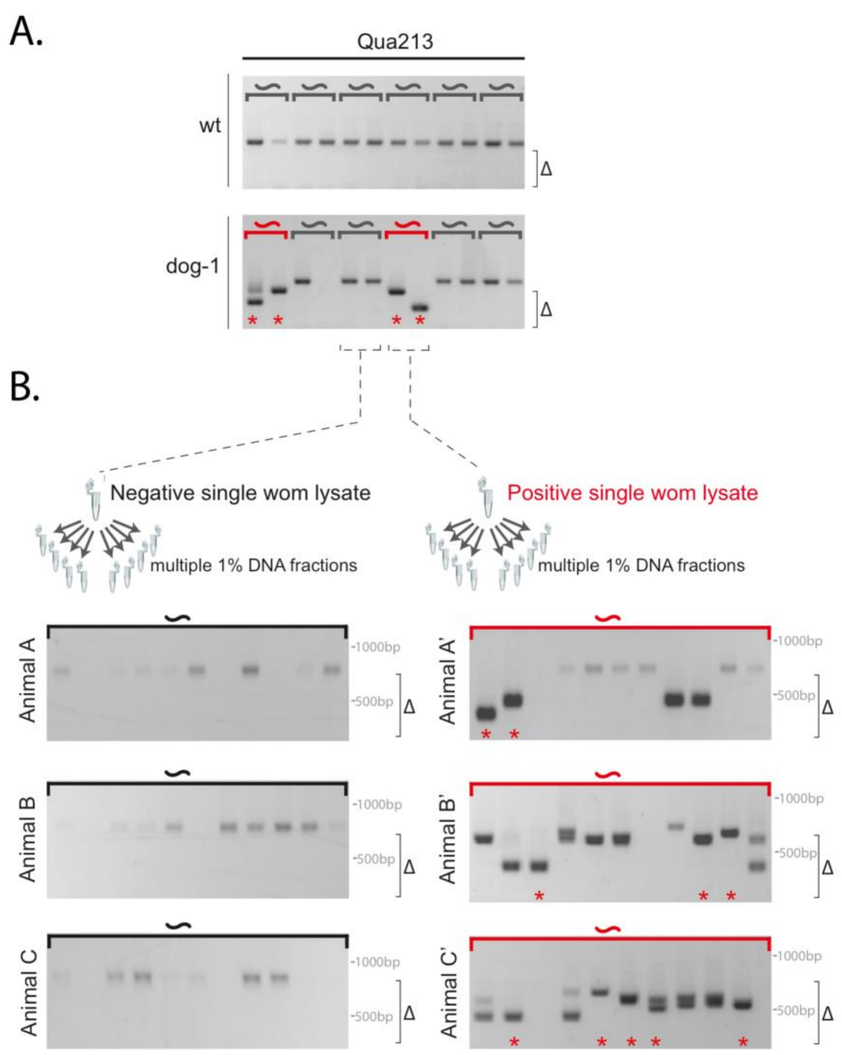


**Supplementary Figure 3. Co-occurrence of multiple locus-specific deletions in single animals**

**A.** PCR analysis of G4 instability at endogenous G4 site Qua213 in wild-type and *dog-1* animals, upper and lower panel, respectively; each well represents an independent PCR reaction on 1% single worm lysate; size-range of PCR-amplified deletions products is indicated by Δ. **B.** Single worm lysates were first categorized based on the presence or absence of G4 deletions in two trial PCRs and subsequently assayed eleven times to probe for the presence of additional G4 deletions. Three gel images representative for each category are depicted. The size-range of PCR-amplified deletions products is indicated by Δ; two reference size markers (500bp and 1000bp) are indicated; red asterisks mark positive reactions.

**Supplementary Figure 4**

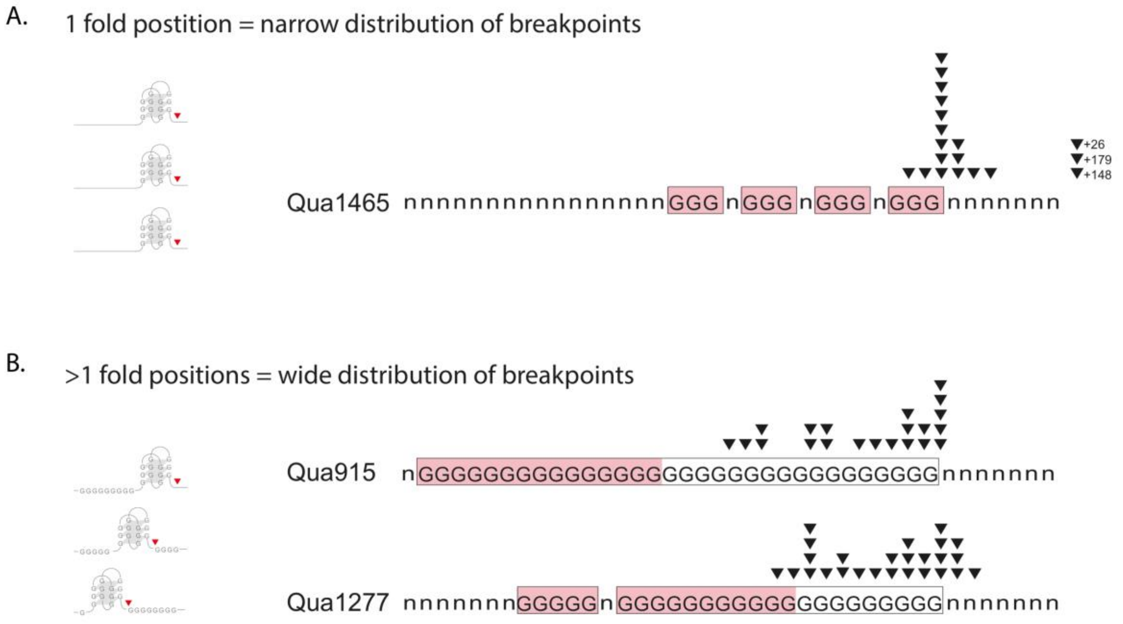


**Supplementary Figure 4. Deletion signatures of G-quadruplex configurations**

**A.** Illustrations on the left portray potential *in vivo* G-quadruplex configurations at a minimal G4 motif such as Qua1465. On the right, the spectrum of 3′ deletion junctions found at Qua1465 (idem as Figure 3B). Each black triangle represents a 3′ deletion junction independently derived from single animals. **B.** Illustrations on the left portray potential *in vivo* G-quadruplex configurations at G4 motifs akin Qua915 and Qua1277 that have multiple G4 folding possibilities. On the right, the spectra of 3′ deletion junctions found at Qua915 and Qua1277. Each black triangle represents a 3′ deletion junction

**Supplementary Figure 5**

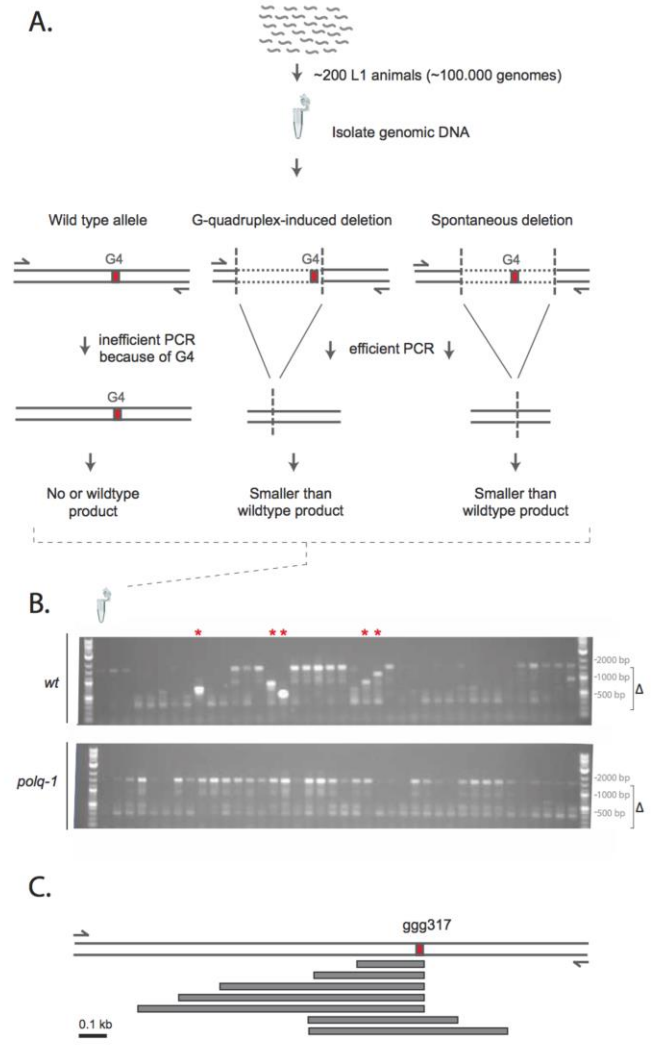


**Supplementary Figure 5. Unidirectional G-quadruplex deletions in wild-type animals**

**A.** Schematic overview of PCR-based experimental setup to identify G4 deletions in wildtype animals using nested PCR reactions on lysates isolated from approximately 200 animals of the larval L1 stage **B.** PCR analysis of G-quadruplex instability at endogenous G4 site Qua1894 in wildtype (upper panel) and *polq-1* deficient animals (lower panel). Each lane represents a different population of 200 animals. Representative gel images are depicted and size-range of PCR-amplified deletions products is indicated by Δ; two reference size markers (500bp and 1000bp) are indicated; red asterisks mark positive reactions. **C.** Size distribution and genomic location of deletions identified in wildtype animals near Qua1894. Only deletions that were repeatedly found by re-assaying the original positive genomic lysate were included. Based on the position of the 3’ junctions, 4 out of 6 deletions constitute typical G-quadruplex-induced deletions. Importantly, it should be noted that PCR-based strategies favor the amplification of small-size bands and thus skew towards the finding of larger deletions, especially in conditions where wild type template is in great excess.

**Supplementary Figure 6**

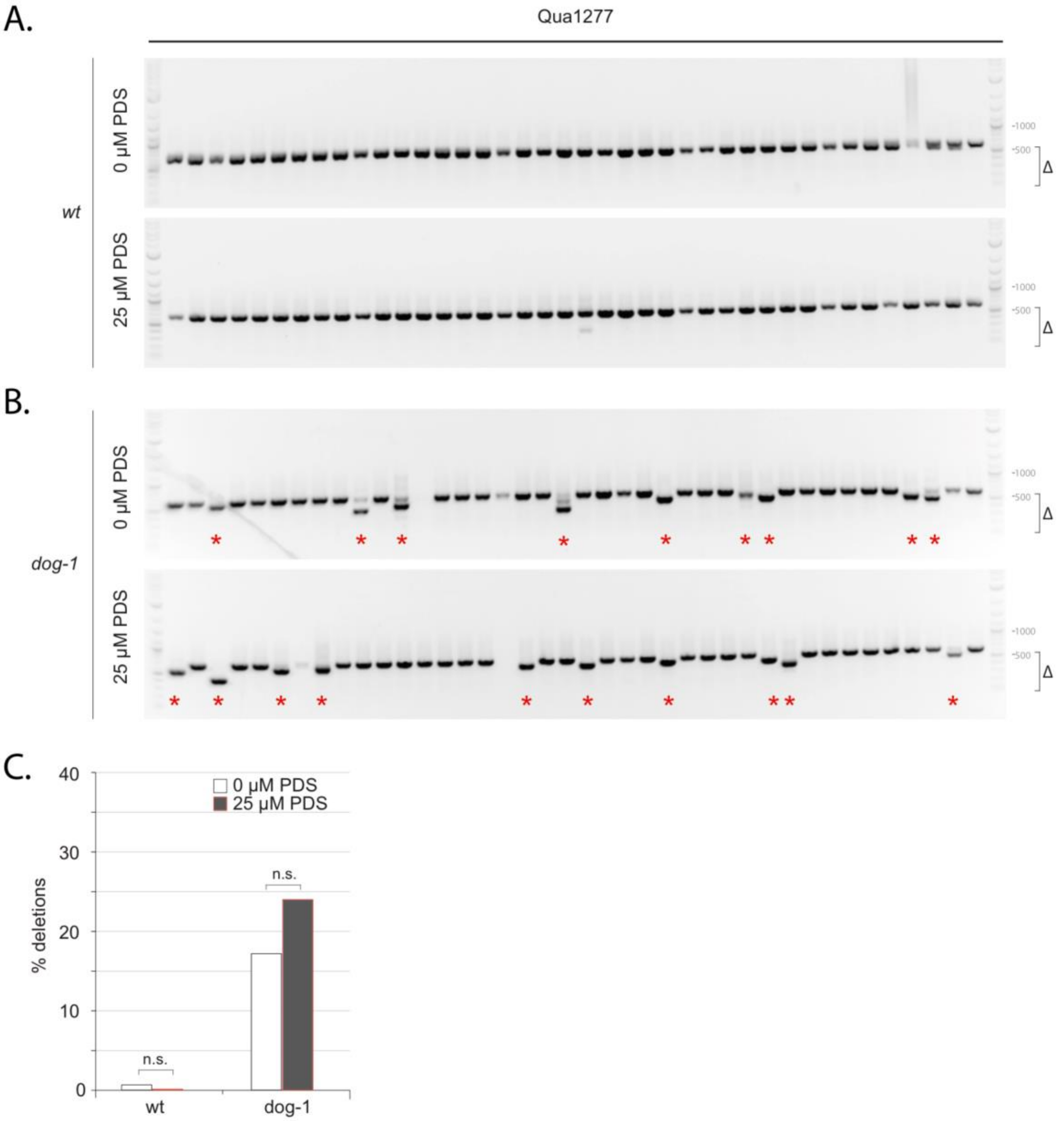


**Supplementary Figure 6. No increase of G4 instability upon chronic Pyridostatin treatment**

PCR analysis of G-quadruplex instability at endogenous G4 site Qua1277 in wildtype (**A**) and *dog-1* deficient (**B**) L4 animals that were exposed to 0 or 25uM Pyridostatin (PDS) for 72 hours; each well represents an independent PCR reaction on 10% single worm lysate; red asterisks mark positive reactions. The size-range of PCR-amplified deletions products is indicated by Δ; two reference size markers (500bp and 1000bp) are indicated. **C.** Quantified deletions frequencies at Qua1277; >140 single worm lysates were analyzed per condition (see **A** and **B** for representative gel images). While chronic treatment with PDS concentrations >50µM resulted in robust developmental arrest, chronic treatment with 25µM PDS allowed L4 larva to develop into fertile adults. In both wildtype and *dog-1* animals, 25µM PDS treatment did not result in a significant increase in deletion formation (p>0.15 by Fisher's exact test, two tailed).

**Supplementary Figure 7**

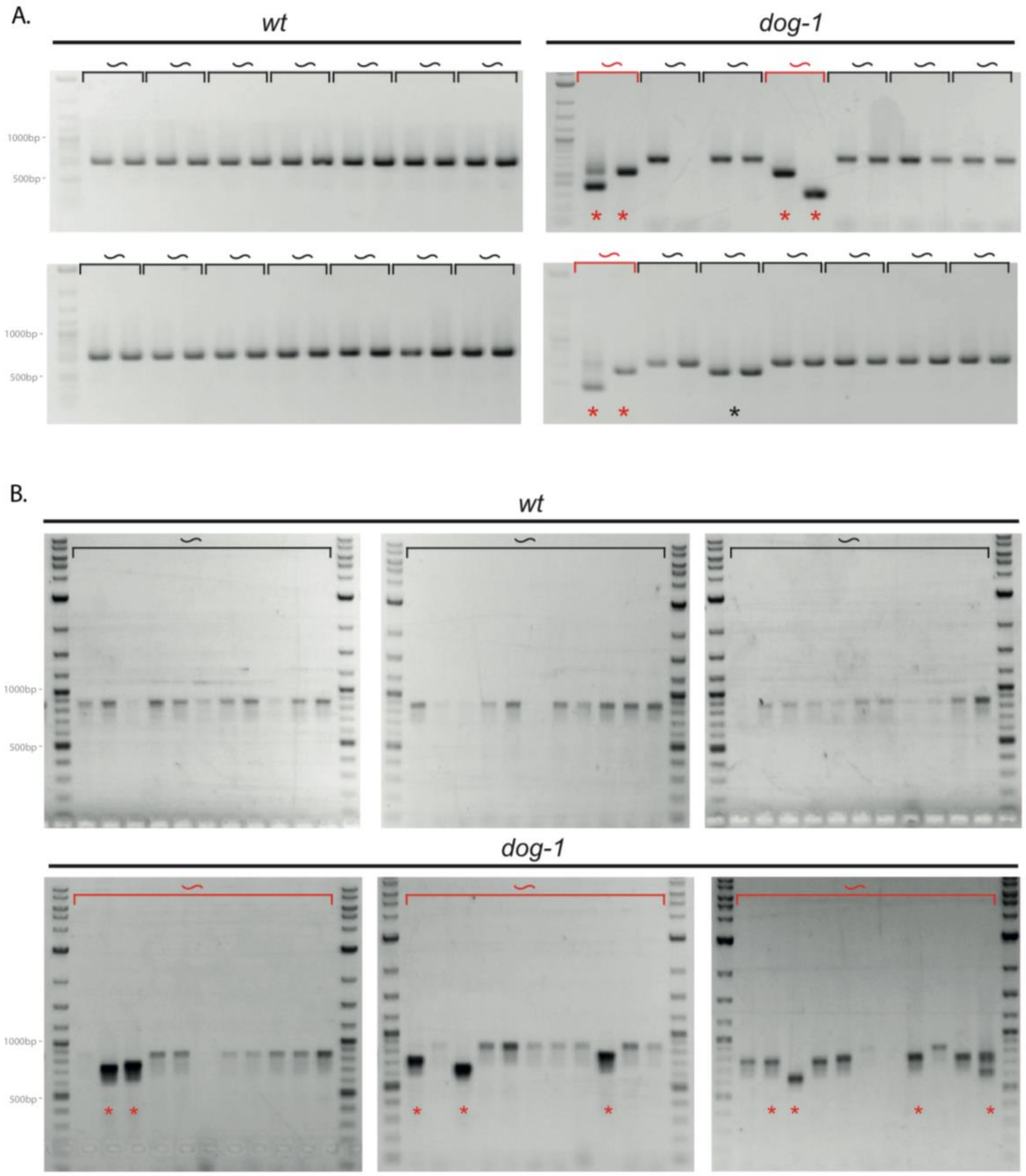


**Supplementary Figure 7. PCR analysis of co-occurring G4 deletions in single animals**

**A.** PCR analysis of G-quadruplex-induced instability at endogenous G4 site Qua1277 in *dog-1* proficient and deficient animals; Representative gel images display the result of single animals (~) assayed twice independently. Reference size markers (500bp and 1000bp) are indicated; asterisks mark unique deletion products and samples having >1 unique deletion are highlighted in red. **B.** Uncropped images of gel lanes depicted in Figure 2D, including 2-log DNA ladder (NEB). Reference size markers (500bp and 1000bp) are indicated.

**Supplementary references**

1. D. B. Pontier, E. Kruisselbrink, V. Guryev, M. Tijsterman, Isolation of deletion alleles by G4 DNA-induced mutagenesis. *Nat Methods* 6, 655 (Sep, 2009).
2. E. Kruisselbrink *et al.*, Mutagenic capacity of endogenous G4 DNA underlies genome instability in FANCJ-defective C. elegans. *Curr Biol* 18, 900 (Jun 24, 2008).
3. C. Frokjaer-Jensen *et al.*, Single-copy insertion of transgenes in Caenorhabditis elegans. *Nature genetics* 40, 1375 (Nov, 2008).
4. J. Pothof *et al.*, Identification of genes that protect the C. elegans genome against mutations by genome-wide RNAi. *Genes Dev* 17, 443 (Feb 15, 2003).
